# 1-(4-Methoxy­phen­yl)imidazolidine-2,4-dione

**DOI:** 10.1107/S1600536810016478

**Published:** 2010-05-12

**Authors:** Su-Xia Sun, Hao Zhang, Xian-Chao Cheng, Run-Ling Wang, Wei-Li Dong

**Affiliations:** a School of Pharmacy, Tianjin Medical University, Tianjin 300070, People’s Republic of China

## Abstract

In the title compound, C_10_H_10_N_2_O_3_, the dihedral angle between the benzene and imidazolidine rings is 6.0 (4)°, consistent with an essentially planar mol­ecule. In the crystal, inter­molecular N—H⋯O hydrogen bonding between centrosymmetrically related mol­ecules leads to loosely associated dimeric aggregates. These are connected into a three-dimensional network by C—H⋯O inter­actions, as well as π–π inter­actions [centroid–centroid distances = 3.705 (3) and 3.622 (3) Å] between the imidazolidine and benzene rings.

## Related literature

For related structures, see: Gerdil (1960 ▶). For the synthesis, see: Niwata *et al.* (1997 ▶); Kurzer *et al.* (1963 ▶).
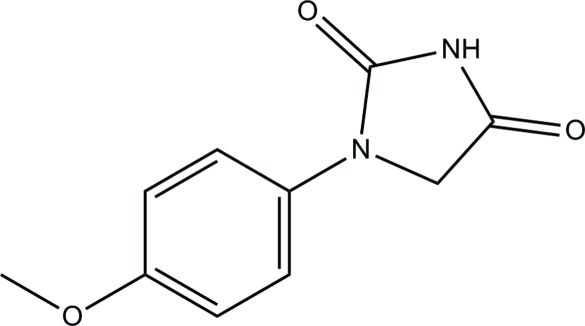


## Experimental

### 

#### Crystal data

C_10_H_10_N_2_O_3_
*M* *_r_* = 206.20
Monoclinic, 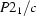

*a* = 4.9993 (10) Å
*b* = 6.1566 (12) Å
*c* = 30.052 (6) Å
β = 93.91 (3)°
*V* = 922.8 (3) Å^3^
*Z* = 4
Mo *K*α radiation
μ = 0.11 mm^−1^
*T* = 113 K
0.24 × 0.12 × 0.10 mm

#### Data collection

Rigaku Saturn CCD area-detector diffractometer
Absorption correction: multi-scan (*CrystalClear*; Rigaku/MSC, 2005 ▶) *T*_min_ = 0.974, *T*_max_ = 0.989
6955 measured reflections
2203 independent reflections
1507 reflections with *I* > 2σ(*I*)
*R*_int_ = 0.078

#### Refinement

*R*[*F* ^2^ > 2σ(*F* ^2^)] = 0.064
*wR*(*F* ^2^) = 0.151
*S* = 1.05
2203 reflections
142 parameters
1 restraint
H atoms treated by a mixture of independent and constrained refinement
Δρ_max_ = 0.44 e Å^−3^
Δρ_min_ = −0.40 e Å^−3^

### 

Data collection: *CrystalClear* (Rigaku/MSC, 2005 ▶); cell refinement: *CrystalClear*; data reduction: *CrystalClear*; program(s) used to solve structure: *SHELXTL* (Sheldrick, 2008 ▶); program(s) used to refine structure: *SHELXTL*; molecular graphics: *SHELXTL*; software used to prepare material for publication: *SHELXTL*.

## Supplementary Material

Crystal structure: contains datablocks I, global. DOI: 10.1107/S1600536810016478/tk2669sup1.cif

Structure factors: contains datablocks I. DOI: 10.1107/S1600536810016478/tk2669Isup2.hkl

Additional supplementary materials:  crystallographic information; 3D view; checkCIF report

## Figures and Tables

**Table 1:** Hydrogen-bond geometry (Å, °)

*D*—H⋯*A*	*D*—H	H⋯*A*	*D*⋯*A*	*D*—H⋯*A*
N1—H1⋯O1^i^	0.91 (1)	1.95 (1)	2.8512 (19)	172 (2)
C2—H2*A*⋯O2^ii^	0.99	2.34	3.291 (2)	160
C8—H8⋯O2^iii^	0.95	2.42	3.203 (2)	140

Symmetry codes: (i) 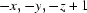; (ii) 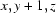; (iii) 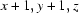.

**Fig. 1.**
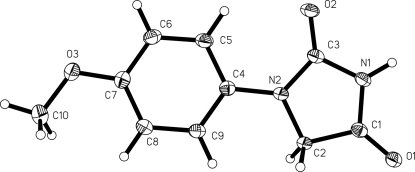
View of the title compound showing atom labelling, with displacement ellipsoids drawn at the 40% probability level.

## supplementary crystallographic information

### Comment

During an investigation of new anti-diabetic drugs, we found that imidazolidinediones (IZD's) have good anti-diabetic activities. The crystal structure determination of the title compound, (I), was undertaken to investigate the relationship between structure and anti-diabetic activity.

In title compound, C_10_H_10_N_2_O_3_, bond lengths and angles are normal and in a good agreement with those reported previously (Gerdil, 1960). The dihedral angle between the benzene ring (C4—C9) and imidazolidine ring (C1—C3/N1/N2) is 6.0 (4) °. In the crystal packing, intermolecular N—H···O hydrogen bonding between centrosymmetrically related molecules lead to loosely associated dimeric aggregates, Table 1. These aggregates are connected into the 3-D crystal structure by C—H···O and π–π interactions, the latter occurring between the imidazolidine and benzene rings, Table 1.

### Experimental

Compound (I) (1.13 g, 55% yield) was prepared according to the reported procedure of (Niwata *et al.*, 1997), using 1-(4-methoxyphenyl)urea (0.010 mol; Kurzer *et al.*, 1963), sodium hydride (0.022 mol), *N*,*N*-dimethylformamide (20 ml), and ethyl chloroacetate (0.0120 mol. Colourless single crystals suitable for X-ray diffraction analysis were obtained by recrystallization from a mixture of methanol and water (1:1 V/V).

### Refinement

All C-bound H atoms were found on difference maps, but included in the final cycles of refinement using a riding model with C—H = 0.95–0.99 Å, and with U_iso_(H) = 1.2U_eq_(C) for aryl- and methylene-H atoms, and 1.5U_eq_(C) for the methyl H atoms. The N–H1 atom was refined freely.

### Crystal data

**Table:**

C_10_H_10_N_2_O_3_	*F*(000) = 432
*M**_r_* = 206.20	*D*_x_ = 1.484 Mg m^−^^3^
Monoclinic, *P*2_1_/*c*	Mo *K*α radiation, λ = 0.71073 Å
Hall symbol: -P 2ybc	Cell parameters from 196 reflections
*a* = 4.9993 (10) Å	θ = 2.0–27.9°
*b* = 6.1566 (12) Å	µ = 0.11 mm^−^^1^
*c* = 30.052 (6) Å	*T* = 113 K
β = 93.91 (3)°	Prism, colourless
*V* = 922.8 (3) Å^3^	0.24 × 0.12 × 0.10 mm
*Z* = 4	

### Data collection

**Table:**

Rigaku Saturn CCD area-detector diffractometer	2203 independent reflections
Radiation source: rotating anode	1507 reflections with *I* > 2σ(*I*)
multilayer	*R*_int_ = 0.078
Detector resolution: 7.31 pixels mm^-1^	θ_max_ = 27.9°, θ_min_ = 2.7°
ω and φ scans	*h* = −6→4
Absorption correction: multi-scan (*CrystalClear*; Rigaku/MSC, 2005)	*k* = −8→7
*T*_min_ = 0.974, *T*_max_ = 0.989	*l* = −34→39
6955 measured reflections	

### Refinement

**Table:**

Refinement on *F*^2^	Secondary atom site location: difference Fourier map
Least-squares matrix: full	Hydrogen site location: inferred from neighbouring sites
*R*[*F*^2^ > 2σ(*F*^2^)] = 0.064	H atoms treated by a mixture of independent and constrained refinement
*wR*(*F*^2^) = 0.151	*w* = 1/[σ^2^(*F*_o_^2^) + (0.0718*P*)^2^] where *P* = (*F*_o_^2^ + 2*F*_c_^2^)/3
*S* = 1.05	(Δ/σ)_max_ = 0.001
2203 reflections	Δρ_max_ = 0.44 e Å^−^^3^
142 parameters	Δρ_min_ = −0.40 e Å^−^^3^
1 restraint	Extinction correction: *SHELXTL* (Sheldrick, 2008), Fc^*^=kFc[1+0.001xFc^2^λ^3^/sin(2θ)]^-1/4^
Primary atom site location: structure-invariant direct methods	Extinction coefficient: 1.95 (8)

### Special details

**Table:**

Geometry. All esds (except the esd in the dihedral angle between two l.s. planes) are estimated using the full covariance matrix. The cell esds are taken into account individually in the estimation of esds in distances, angles and torsion angles; correlations between esds in cell parameters are only used when they are defined by crystal symmetry. An approximate (isotropic) treatment of cell esds is used for estimating esds involving l.s. planes.
Refinement. Refinement of *F*^2^ against ALL reflections. The weighted *R*-factor wR and goodness of fit *S* are based on *F*^2^, conventional *R*-factors *R* are based on *F*, with *F* set to zero for negative *F*^2^. The threshold expression of *F*^2^ > σ(*F*^2^) is used only for calculating *R*-factors(gt) etc. and is not relevant to the choice of reflections for refinement. *R*-factors based on *F*^2^ are statistically about twice as large as those based on *F*, and *R*- factors based on ALL data will be even larger.

### Fractional atomic coordinates and isotropic or equivalent isotropic displacement parameters (Å^2^)

**Table:**

	*x*	*y*	*z*	*U*_iso_*/*U*_eq_	
O1	0.1590 (2)	0.2744 (2)	0.50596 (4)	0.0270 (4)	
O2	0.4023 (2)	−0.2177 (2)	0.40235 (4)	0.0286 (4)	
O3	1.2460 (3)	0.2947 (2)	0.28363 (4)	0.0303 (4)	
N1	0.2393 (3)	−0.0069 (2)	0.45821 (5)	0.0232 (4)	
N2	0.5536 (3)	0.1341 (2)	0.41800 (5)	0.0217 (4)	
C1	0.2749 (3)	0.1953 (3)	0.47530 (6)	0.0221 (4)	
C2	0.4866 (3)	0.3027 (3)	0.44955 (5)	0.0213 (4)	
H2A	0.4155	0.4340	0.4338	0.026*	
H2B	0.6448	0.3433	0.4694	0.026*	
C3	0.4027 (3)	−0.0471 (3)	0.42284 (6)	0.0221 (4)	
C4	0.7341 (3)	0.1737 (3)	0.38439 (5)	0.0209 (4)	
C5	0.7790 (3)	0.0195 (3)	0.35134 (6)	0.0265 (4)	
H5	0.6900	−0.1168	0.3514	0.032*	
C6	0.9526 (3)	0.0659 (3)	0.31876 (6)	0.0272 (5)	
H6	0.9814	−0.0391	0.2964	0.033*	
C7	1.0854 (3)	0.2637 (3)	0.31826 (6)	0.0236 (4)	
C8	1.0464 (3)	0.4155 (3)	0.35116 (6)	0.0244 (4)	
H8	1.1385	0.5505	0.3513	0.029*	
C9	0.8710 (3)	0.3692 (3)	0.38415 (6)	0.0230 (4)	
H9	0.8452	0.4735	0.4068	0.028*	
C10	1.3963 (4)	0.4914 (3)	0.28380 (7)	0.0339 (5)	
H10A	1.2732	0.6153	0.2811	0.051*	
H10B	1.5108	0.4915	0.2586	0.051*	
H10C	1.5082	0.5027	0.3118	0.051*	
H1	0.113 (3)	−0.100 (3)	0.4673 (7)	0.045 (6)*	

### Atomic displacement parameters (Å^2^)

**Table:**

	*U*^11^	*U*^22^	*U*^33^	*U*^12^	*U*^13^	*U*^23^
O1	0.0294 (7)	0.0210 (8)	0.0312 (7)	−0.0022 (5)	0.0070 (5)	−0.0019 (6)
O2	0.0365 (7)	0.0170 (8)	0.0321 (7)	−0.0036 (5)	0.0020 (5)	−0.0035 (5)
O3	0.0331 (7)	0.0295 (9)	0.0295 (7)	−0.0001 (5)	0.0103 (5)	−0.0008 (6)
N1	0.0260 (7)	0.0171 (9)	0.0264 (8)	−0.0035 (5)	0.0012 (6)	0.0003 (6)
N2	0.0269 (8)	0.0151 (8)	0.0232 (8)	−0.0022 (5)	0.0035 (6)	−0.0020 (6)
C1	0.0243 (8)	0.0183 (10)	0.0231 (8)	−0.0002 (6)	−0.0027 (6)	0.0020 (7)
C2	0.0263 (8)	0.0152 (9)	0.0226 (9)	−0.0019 (6)	0.0031 (6)	−0.0019 (7)
C3	0.0253 (8)	0.0171 (10)	0.0235 (9)	−0.0010 (6)	−0.0022 (6)	0.0001 (7)
C4	0.0226 (8)	0.0184 (10)	0.0212 (8)	0.0021 (6)	−0.0006 (6)	0.0015 (7)
C5	0.0320 (9)	0.0181 (10)	0.0295 (10)	−0.0007 (7)	0.0022 (7)	−0.0018 (7)
C6	0.0339 (10)	0.0229 (10)	0.0249 (9)	0.0025 (7)	0.0026 (7)	−0.0038 (8)
C7	0.0219 (9)	0.0259 (11)	0.0229 (9)	0.0041 (6)	0.0011 (6)	0.0022 (7)
C8	0.0256 (9)	0.0216 (10)	0.0259 (9)	−0.0029 (6)	−0.0004 (7)	0.0004 (7)
C9	0.0267 (9)	0.0193 (10)	0.0226 (8)	−0.0008 (6)	−0.0001 (6)	−0.0020 (7)
C10	0.0310 (10)	0.0359 (13)	0.0353 (11)	−0.0048 (8)	0.0067 (8)	0.0036 (9)

### Geometric parameters (Å, °)

**Table:**

O1—C1	1.223 (2)	C4—C9	1.385 (2)
O2—C3	1.217 (2)	C4—C5	1.403 (2)
O3—C7	1.370 (2)	C5—C6	1.382 (2)
O3—C10	1.425 (2)	C5—H5	0.9500
N1—C1	1.354 (2)	C6—C7	1.388 (3)
N1—C3	1.406 (2)	C6—H6	0.9500
N1—H1	0.911 (10)	C7—C8	1.384 (2)
N2—C3	1.360 (2)	C8—C9	1.397 (2)
N2—C4	1.420 (2)	C8—H8	0.9500
N2—C2	1.460 (2)	C9—H9	0.9500
C1—C2	1.506 (2)	C10—H10A	0.9800
C2—H2A	0.9900	C10—H10B	0.9800
C2—H2B	0.9900	C10—H10C	0.9800
			
C7—O3—C10	116.85 (14)	C6—C5—C4	120.02 (17)
C1—N1—C3	112.37 (14)	C6—C5—H5	120.0
C1—N1—H1	122.9 (15)	C4—C5—H5	120.0
C3—N1—H1	124.5 (15)	C5—C6—C7	120.87 (17)
C3—N2—C4	126.92 (15)	C5—C6—H6	119.6
C3—N2—C2	111.09 (14)	C7—C6—H6	119.6
C4—N2—C2	121.66 (14)	O3—C7—C8	124.53 (17)
O1—C1—N1	126.52 (17)	O3—C7—C6	115.84 (16)
O1—C1—C2	126.75 (17)	C8—C7—C6	119.62 (16)
N1—C1—C2	106.72 (15)	C7—C8—C9	119.67 (17)
N2—C2—C1	102.84 (14)	C7—C8—H8	120.2
N2—C2—H2A	111.2	C9—C8—H8	120.2
C1—C2—H2A	111.2	C4—C9—C8	121.02 (16)
N2—C2—H2B	111.2	C4—C9—H9	119.5
C1—C2—H2B	111.2	C8—C9—H9	119.5
H2A—C2—H2B	109.1	O3—C10—H10A	109.5
O2—C3—N2	129.43 (17)	O3—C10—H10B	109.5
O2—C3—N1	123.60 (16)	H10A—C10—H10B	109.5
N2—C3—N1	106.95 (14)	O3—C10—H10C	109.5
C9—C4—C5	118.78 (16)	H10A—C10—H10C	109.5
C9—C4—N2	119.40 (15)	H10B—C10—H10C	109.5
C5—C4—N2	121.82 (16)		
			
C3—N1—C1—O1	179.61 (16)	C3—N2—C4—C5	−0.8 (3)
C3—N1—C1—C2	−0.94 (18)	C2—N2—C4—C5	−173.66 (15)
C3—N2—C2—C1	1.19 (18)	C9—C4—C5—C6	−1.4 (2)
C4—N2—C2—C1	175.05 (14)	N2—C4—C5—C6	178.85 (15)
O1—C1—C2—N2	179.32 (16)	C4—C5—C6—C7	0.3 (3)
N1—C1—C2—N2	−0.13 (17)	C10—O3—C7—C8	4.6 (2)
C4—N2—C3—O2	6.1 (3)	C10—O3—C7—C6	−176.52 (15)
C2—N2—C3—O2	179.53 (17)	C5—C6—C7—O3	−178.02 (15)
C4—N2—C3—N1	−175.22 (14)	C5—C6—C7—C8	0.9 (3)
C2—N2—C3—N1	−1.77 (19)	O3—C7—C8—C9	177.92 (14)
C1—N1—C3—O2	−179.49 (15)	C6—C7—C8—C9	−0.9 (2)
C1—N1—C3—N2	1.71 (19)	C5—C4—C9—C8	1.4 (3)
C3—N2—C4—C9	179.43 (16)	N2—C4—C9—C8	−178.85 (14)
C2—N2—C4—C9	6.6 (2)	C7—C8—C9—C4	−0.3 (3)

### Hydrogen-bond geometry (Å, °)

**Table:**

*D*—H···*A*	*D*—H	H···*A*	*D*···*A*	*D*—H···*A*
N1—H1···O1^i^	0.91 (1)	1.95 (1)	2.8512 (19)	172 (2)
C2—H2A···O2^ii^	0.99	2.34	3.291 (2)	160
C8—H8···O2^iii^	0.95	2.42	3.203 (2)	140
Cg1—···.Cg1^iv^	.	.	3.705 (3)	.
Cg1—···.Cg2^v^	.	.	3.622 (3)	.

Symmetry codes: (i) −*x*, −*y*, −*z*+1; (ii) *x*, *y*+1, *z*; (iii) *x*+1, *y*+1, *z*; (iv) −*x*+1, −*y*, −*z*; (v) *x*+1, *y*, *z*.
